# Attitudes toward veganism in eating disorder professionals

**DOI:** 10.1192/bjb.2021.57

**Published:** 2022-04

**Authors:** Sarah J. Fuller, Kimberley M. Hill

**Affiliations:** 1 Bedfordshire & Luton CAMHS Eating Disorders Team, East London NHS Foundation Trust, UK; 2 Faculty of Health, Education and Society, University of Northampton, UK

**Keywords:** Eating disorders, veganism, stigma and discrimination, in-patient treatment, ethics

## Abstract

**Aims and method:**

Veganism has increased in popularity in the past decade and, despite being a characteristic protected by law, is often viewed negatively by the general population. Little is known about the attitudes of healthcare professionals despite the potential influence on practice and eating disorder patient care. This is one of the first studies to investigate attitudes toward veganism within specialist eating disorder, general mental health and other professionals.

**Results:**

A one-way ANOVA indicated all professionals held positive views toward veganism. General mental health professionals held statistically more positive veganism attitudes than specialist eating disorder and other professionals.

**Clinical implications:**

As one of the first studies to suggest eating disorder professionals are not biased against veganism, it has important clinical practice implications, particularly when exploring motivations for adopting a vegan diet (health, weight loss, environmental or animal welfare concerns) in patients with eating disorders. Implications for further research are provided.

Eating disorders are serious psychiatric conditions characterised by abnormal eating patterns, either through strict or a lack of control of eating, and are driven by the over-evaluation of weight and shape concerns.^1^ Research identifies several eating disorder development risk factors,^2^ including, but not limited to, genetics,^3,4^ environmental,^3^ female adolescence,^4^ biopsychosocial influences^5^ and urbanisation.^6^

Veganism is a philosophy seeking to exclude using animals and animal products in all aspects of life, not just diet.^7^ It is estimated that around 1% of the UK population follow a vegan diet,^8^ reflecting a fourfold increase from 2014 to 2019. In the Western world, the demographics of veganism are predominantly young,^9^ female^10,11^ and living in urban areas.^10,11^ Importantly, veganism is protected by law as a non-religious philosophical belief.^12^

Veganism does not cause eating disorders, but there are similarities between known eating disorder risk factors and the prevalence data for veganism. Research suggests the general population are biased against veganism,^13,14^ but it remains unknown if specialist eating disorder (SED) professionals share these views. SED clinicians may be concerned about the potentially restrictive nature of vegan diets, and therefore may potentially be biased against veganism. Given the legal protection that veganism carries, when exploring the veganism of a patient with an eating disorder, clinicians must act with professionalism, respect, curiosity and a lack of personal bias. This is the first paper to investigate the attitudes of SED, general mental health (GMH) and other professionals toward veganism

## Method

### Design

This self-reported questionnaire study included the following independent variables: profession (SED, GMH and other professionals), age (18–29, 30–41, 42–54 and 55–67 years) and gender (male, female and other). The dependent variable was attitude toward veganism.

### Pilot study

A pilot study involving three participants from SED, GMH and other professional groups indicated no adjustments to study measures.

### Measures

Participants completed the General Eating Habits Questionnaire,^15^ scored on a 1 (vegan) to 7 (omnivore) Likert scale, and the 20-question Attitudes Towards Veganism Survey (ATvegan),^10^ scored on a 1 (negative attitudes) to 7 (positive attitudes) Likert scale, on Qualtrics (version for Windows; www.qualtrics.com). Cronbach's alpha (*α* = 0.86) indicated a good level of internal reliability.^11^

### Participants and recruitment

A power analysis (G*Power for Windows, version 3.1.9.7; https://www.psychologie.hhu.de/arbeitsgruppen/allgemeine-psychologie-und-arbeitspsychologie/gpower.html) identified *n* = 75 for each professional group, giving a minimum sample size of 225. Participants were recruited through purposive sampling via professional networks and social media. A total of 430 responses were received and data were cleaned to exclude non-UK residents (*n* = 15), those under 18 or older than 68 years, those not identifying their professional group (*n* = 3) and vegan participants (*n* = 20), as research suggested this could bias responses as vegans have an strong sense of self-identity, which can affect their attitudes on topics ranging from animal welfare to political affiliation, and this could affect any findings of the research.^13,16^ Table 1 provides key characteristics of the total sample (*n =* 392).

**Table 1:** Participant demographics

	Gender, *n*		
Profession	Age group, year	Male	Female	Did not say
Specialist eating disorder, *n* = 116	18–29	2	14	--
	30–41	7	33	--
	42–54	15	25	--
	55–68	5	15	--
General mental health, *n* = 90	18–29	4	18	--
	30–41	7	31	1
	42–54	5	15	1
	55–68	6	2	--
Other, *n* = 186	18–29	8	32	--
	30–41	26	55	--
	42–54	14	28	--
	55–68	8	15	--

### Procedure

Full ethical approval was obtained and, following written informed consent, data was collected for 2 weeks during March 2020. Following completion, participants were debriefed, thanked and provided with researcher contact details for further questions. Ethical approval was granted by the University of Northampton's Psychology Ethics Committee (ethics approval donated by student number: 19432991). All adult participants provided written informed consent to participate in this study.

### Analysis

Data was analysed using SPSS version 26 for Windows.

## Results

An alpha level of 0.05 was used for all statistical tests.

### Profession

Total attitude toward veganism scores were calculated indicating generally high mean scores and positive attitudes for all professional groups. This included the GMH (*n* = 90, mean 106.65, s.d. 17.96, range 54–137), SED (*n* = 116, mean 101.49, s.d. 16.13, range 61–136) and other professionals groups (*n* = 186, mean 101.08, s.d. 18.64, range 43–140).

All parametric assumptions were met. A one-way ANOVA was statistically significant, indicating a moderate effect size and a positive main effect of professional group (*F*(2, 376) = 3.33, *P* = 0.04, *η_p_*^2^ = 0.02).

*Post hoc* Bonferroni adjustments^14^ indicated mean GMH professionals group scores (mean 106.65 ± 5.72, s.d. 17.96, *P* = 0.04) were significantly higher and more positive compared with the other professionals group. No significant difference was evident between the SED and GMH or other professionals groups.

### Gender

Women (*n =* 290, mean 103.36, s.d. 19.24, range 59–140) had slightly higher mean veganism attitude scores than men (*n* = 100, mean 99.95, s.d. 17.33, range 43–134), but a Mann–Whitney *U*-test conducted on non-parametric data indicated no significant difference between women (median = 104, *n* = 279) and men (median = 101.5, *n* = 98) and attitude toward veganism scores (*U* = 14 777.00, *z* = 1.19, *P* = 0.23).

### Age

Younger participants aged 18–29 years had higher mean and more positive attitudes toward vegan scores (*n* = 78, mean 104.48, s.d. 16.74, range 66–138), compared with ages 30–41 years (*n* = 163, mean 102.17, s.d. 18.56, range 43–137), 42–54 years (*n* = 103, mean 102.81, s.d. 16.30, range 69–140) and 55–68 years (*n* = 48, mean 99.44, s.d. 20.40, range 54–135). However, a one-way ANOVA indicated no significant difference between participants’ age and their attitude toward veganism score (*P* = 0.50).

## Discussion

This study is one of the first to investigate veganism attitudes within SED, GMH and other professional groups. The aim was to identify whether the potential bias toward veganism found within the general population is prevalent within SED professionals. Findings suggested all three professional groups held positive veganism attitudes, with GMH professionals holding significantly more positive attitudes than SED and other professionals. Despite age and gender influencing veganism attitudes in the general population, no statistically significant age or gender differences were found within these professional groups.

Research has highlighted a level of bias against veganism within Western populations,^13,17,18^ leading to it being viewed as a minority group similar to ethnicity or sexual orientation.^18^ Not only are vegans often depicted as going against the status quo of normal dietary culture, but these attitudes are influenced by gender and age, with more prominent negative attitudes often found in older, male generations. As well as investigating whether these biases exist within SED professional populations, it was hypothesised that SED professionals would have a more negative view on veganism than other professionals. This is because SED professionals are aware of how dietary restrictions can negatively affect an individual's physical health and mental health. These general attitudes could be reflected in SED professionals’ own veganism attitudes, and SED professionals should be aware of any such biases, as they could affect clinical practice and patient treatment. In 2019, a joint consensus statement from the Royal College of Psychiatrists, the British Dietetic Association and ‘BEAT’, the national eating disorder charity, was released regarding the importance of working collaboratively with vegan patients with eating disorders.^19^ This sought to address concerns raised by some vegan patients that their beliefs were ignored in treatment and that staff could be biased against veganism. In contrast, the current study appears to indicate that SED professionals are not biased toward veganism.

These findings are particularly important because SED professionals may be concerned when patients presenting with eating disorders make any significant dietary change before seeking treatment. Self-imposed dietary restrictions are common in patients with restrictive eating disorders. These restrictions can be total caloric restriction, but can also involve excluding entire food groups such as carbohydrates or fats, or excluding ingredients in foods such as lactose or gluten. It is not uncommon to see numerous, escalating self-imposed dietary restrictions as a patient's eating disorder progresses. For example, someone who previously ate a diet that included meat could become pescatarian, then vegetarian and finally vegan – with each dietary change becoming more restrictive. There is evidence that there are increased rates of vegetarianism in patients with restrictive eating disorders, such as anorexia nervosa.^20,21^ As veganism requires more dietary restrictions than vegetarianism, researchers suggest that a similar link could be associated with veganism,^22^ which could raise concerns for SED professionals. Furthermore, SED professionals will be aware of the overlap in the demographics of veganism and factors that make an individual more susceptable to an eating disorder.

To adopt a vegan diet, an individual must avoid all animal products, ingredients or derivatives. Therefore, this requires them to check dietary labels and will result in the exclusion of foods they previously ate. These two behaviours, checking labels and food exclusion based on ingredients, are often seen in patients with restrictive eating disorders regardless of their overall dietary choice. These firm dietary rules veganism provides can be very attractive to patients who are anxious regarding what to eat. Based on the findings of the current study above, SED professionals do not show a bias against veganism. However, as research suggests that the general population perceive multiple barriers to switching to a vegan diet,^23^ SED clinicians may therefore be suspicious of the apparent coincidence of such a dietary change during the onset of the eating disorder.

### Implications for practice

Findings from this research suggest that SED professionals do not have more negative views on veganism compared with GMH and other professionals. Instead, all groups held positive attitudes toward veganism, with GMH professionals statistically holding the most positive views. This finding may be partly mediated by participant demographics, as GMH professional participants tended to be younger women compared with SED and other professionals. Knowing that SED professionals did not have a negative attitude toward veganism is important because when exploring a patient's veganism, the patient may feel vulnerable having a clinician challenge behaviour that may or may not be associated with their eating disorder. SED professionals can use this research to reassure patients that it is their eating disorder that is being questioned and not their veganism. Taking this dynamic further, it is important for these professionals to be aware of their ‘social GRACES’.^24^ This acronym was developed for clinicians to be aware of the many areas in life where we may have conscious or unconscious bias in clinical work. Using this acronym, there is more than one topic within each ‘letter’ and the full acronym is ‘GGRRAAACCEEESSS’, encompassing gender, geography, race, religion, age, ability, appearance, class, culture, ethnicity, education, employment, sexuality, sexual orientation and spirituality.^24^ Clinicians have both an ethical and legal responsibility to their patients not to bring any bias into the treatments they offer, if they themselves have different dietary choices from their patients,^18,24,25^ so there could be a D added to the ‘social GRACES’ – that of diet and dietary choice.

This research also has a much broader impact as it also reflects that SED professionals are practicing within relevant legal frameworks. As veganism is a protected characteristic within the law, these finds are important.^12,26^ If the main hypothesis of this study had been supported, it would have raised concerns that these professionals’ opinions were significantly different. Going forward, our findings highlight the need for all SED clinicians to have an awareness of the nuanced issues veganism can bring for a patient with an eating disorder, as well as an awareness of the legal protection this characteristic holds. Navigating this difficult dynamic may be helped by this research, as it is one of the first studies to consider these issues.

These findings can be generalised to the wider UK SED profession, and will inform daily clinical practice, particularly as veganism is becoming more popular nationally.^27^ The good response rate and high completion rates suggest that veganism is a topic of interest for professionals. Further international research could help generalise these findings in the wider Western world and globally.

### Limitations

Bias was minimised by using reversed questions, valid instruments and measures, but future research in this area should recognise possibilities for bias. Because of the self-reported nature of this research, participants may have shown demand characteristics (participants changing reported behaviours in line with their interpretation of the study) that may have influenced the findings, particularly given the potential implications for SED and GMH professionals. Consequently, participants may have provided what they perceived as the professionally correct answers,^27,28^ or ‘socially desirable’ responses, rather than declaring any strong personal views to the contrary.^29^

### Future research and conclusions

Exploring the sensitive dynamic of veganism and eating disorders would benefit from further research. This includes investigating the extent that vegan clinicians feel that veganism can be used to facilitate dietary restriction in patients with eating disorders. Research targeting vegan SED professionals will provide an unbiased understanding of how veganism may be used to facilitate dietary restriction in patients with eating disorders. Although ethically sensitive, future research could focus on the extent that patients who have recovered from an eating disorder feel that veganism can be used to facilitate dietary restriction in eating disorders. Including SED professionals and patients who have recovered from an eating disorder from a range of demographics, including age, gender and ethnicity, would allow a more culturally diverse interpretation of this topic area.

The current research study was targeted at clinicians who work either in adult, child or adolescent mental health services. These clinicians may have different attitudes toward veganism when they are working with a child or an adult, and future research should consider potential differences here. For example, a 12-year-old girl who is presenting with a restrictive eating disorder and asking to become vegan for animal welfare reasons may evoke concerns from clinicians regarding the authenticity of this dietary change, especially when the nutritional adequacy of the vegan diet can be hard to achieve in this age group because of the nutritional demands of growth and puberty.^30^ In contrast, an adult patient following a vegan diet because of a family history of heart disease may seem less concerning to SED professionals, and this research did not differentiate between the different motivations a patient may have. Therefore, future research should differentiate within the SED group by their area of speciality – children and young people or adult. Theoretically, an age-informed professional consensus could be developed, potentially demonstrating greater concern for younger patients or those who are following an increasing pattern of dietary restrictions leading to veganism.

In conclusion, veganism is an increasing and legally protected characteristic, but the general population hold negative attitudes toward veganism. Research identifies important similarities between vegan demographics and those at risk of developing an eating disorder. As a patient's veganism may be challenged as part of their eating disorder treatment, it is important to identify if SED professionals hold the same biases, as this could have important implications for patient care and practice. This is the first study to highlight that SED professionals do not appear to be biased; in contrast, they hold positive views toward veganism, as do GMH and other professionals. Not only does this research suggest that SED professionals are practicing within the law, but it also indicates that they are aware of their social GRACES, and perhaps a ‘D’ for ‘diet’ could be added to this acronym. Further in-depth and more diverse research into professional's attitudes toward veganism is encouraged.

## About the authors

**Sarah J. Fuller** is an Advanced Specialist Eating Disorders Dietitian with the Bedfordshire & Luton CAMHS Eating Disorders Team, East London NHS Foundation Trust, UK. **Kimberley M. Hill** is an Associate Professor of Psychology with the Faculty of Health, Education and Society, University of Northampton, UK.

## Data availability

The data that support the findings of this study are available from the corresponding author, S.J.F., upon reasonable request.
